# Opportunities for collaboration: the synergy between antimicrobial and diagnostic stewardship in pediatrics

**DOI:** 10.1017/ash.2024.464

**Published:** 2025-02-07

**Authors:** Brandy M. Hoyt, Kevin Messacar, Anna C. Sick-Samuels, Preeti Jaggi, Stacey L. Hamilton, Sarah Janelle, Sarah K. Parker

**Affiliations:** 1 Department of Pediatrics, Section of Hospital Medicine, University of Colorado School of Medicine, Aurora, CO, USA; 2 Section of Infectious Diseases, Children’s Hospital Colorado, University of Colorado School of Medicine, Aurora, CO, USA; 3 Department of Pediatrics, Johns Hopkins University School of Medicine, Baltimore, MD, USA; 4 Department of Hospital Epidemiology and Infection Control, Johns Hopkins Hospital, Baltimore MD, USA; 5 Department of Pediatrics, Division of Pediatric Infectious Disease, Children’s Healthcare of Atlanta, Atlanta, GA, USA; 6 Department of Pediatrics, Division of Pediatric Infectious Disease, Emory University School of Medicine, Atlanta, GA, USA; 7 Department of Pathology and Laboratory Medicine, Children’s Hospital Colorado, Aurora, CO, USA; 8 Department of Epidemiology, Children’s Hospital Colorado, Aurora, CO, USA

## Abstract

Advancement of antimicrobial stewardship (AS) programs requires partnership with clinicians, quality assurance teams, and laboratorians. Inevitably, AS programs also practice diagnostic stewardship (DS), as stewards are aptly placed to connect key stakeholders and help steer processes toward higher value care for pediatric patients. In this review, we illustrate five moments of collaboration between stakeholders in the interplay between AS and DS in pediatrics. These moments include (1) Observation, (2) Reflection, (3) Exploration, (4) Enactment and (5) Evaluation. We offer a targeted narrative of examples in current literature using common relatable scenarios (ie, endotracheal aspirates, blood cultures, gastrointestinal samples, and urine testing) including impact on financial and environmental waste.

## Introduction

**Figure 1.**
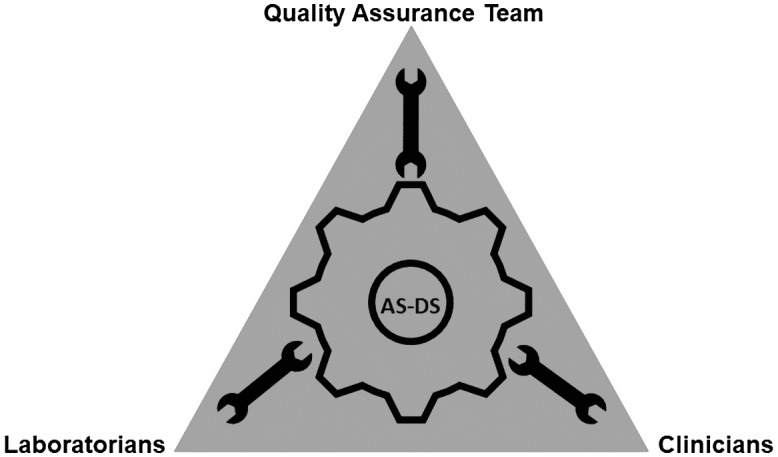
AS-DS synergy: the care triangle between clinicians (eg, physicians, advanced practice providers, pharmacists, nurses), laboratorians, and quality assurance teams (eg, infection preventionists, patient safety specialists, and guideline developers).

Although antimicrobial stewardship (AS) programs guide the judicious use of anti-infectives, as drivers of prescribing are identified, their functionality expands. The optimal AS program evolves to collaborate with clinicians (eg, physicians, advanced practice providers, pharmacists, nurses), laboratorians, and quality assurance teams (eg, infection preventionists, patient safety specialists, and guideline developers) hospital wide, with attention to diagnostic stewardship (DS) to improve care for patients. AS is uniquely centered in the care triangle that connects these stakeholders (Figure 1), though the exact interface will look different based on a hospital’s resources (eg, available testing, on vs off-site microbiology, on vs off-site pharmacists and specialists, AS program presence and design). DS is the process of “modifying the ordering, performing, or reporting of diagnostic tests to improve the diagnosis and treatment of infections and other conditions.”^1^ AS can harness DS and the intrinsic motivation of stakeholders in each corner of the triangle to facilitate collaboration that results in higher value care.

Historically, prior to AS and DS, clinicians ordered tests with minimal laboratory guidance, and the laboratory reported unfiltered results without necessarily including guidance for interpretation. As microbiology testing becomes more advanced, clinical care becomes more complex, and the harms of over-medicalization become more evident, incorporating guidance along the spectrum of testing and treatment benefits patient care. Clinicians are encouraged to weigh pre-test probability so that tests are relevant and actionable, laboratories are empowered to optimize a systematic approach to test implementation and reporting, and quality assurance teams are tasked with identifying targets for improving quality of care, developing guidance, and promoting system improvements.^2^ Infection preventionists and nurses in particular have an underappreciated stake in DS because of the impact testing and results can have on work flow and reportable healthcare-associated infections (HAIs).^3,4^ Depending on local organization, infection prevention and control (IPC) may be part of patient quality/safety, function independently, or directly overlap with antibiotic stewardship roles, and thus share interest and responsibility of DS. AS programs closely interact with all of these stakeholders within a healthcare facility that may be invested to pursue DS and thus are well-positioned to support and partner these initiatives.

The classic elements and concepts of DS are well defined in the literature; these include the pre-analytic (eg, ordering and collection), analytic (eg, laboratory processing), and post-analytic (eg, reporting) phases of testing.^5–8^ In this review, we seek to illustrate these concepts with real-world examples that highlight the creativity and moments of opportunity where stewardship can help steer this synergistic improvement process in pediatrics. These moments include 1) Observation, 2) Reflection, 3) Exploration, 4) Enactment, and 5) Evaluation (Table 1). A few common pediatric scenarios are selected below to exemplify these opportunities in a pragmatic manner.

**Table 1.** Clinical examples of the five moments for AS and DS synergy

5 Moments	Observation: Noticing current low value processes	Reflection: Questioning established systems- “Why do we do it this way? Is this the best process?”	Exploration: Brainstorming better processes, collaborating across multiple disciplines- “Can we add value if we do this differently?”	Enactment: Includes process change and implementation, clinician education, and EMR optimization	Evaluation: Includes assessment of change and balancing measures- “Did these changes add value? Did they cause harm?”
Tracheal aspirates	• Laboratorian: “We do lots of identification and susceptibilities on multiple organisms from poor quality samples.” • AS Team: “Though that result is polymicrobial and a poor sample, people seem to treat any identified bacteria, often with cefepime.” • Clinicians: “The result is shown in red with an exclamation mark, doesn’t that mean it is significant? Should I treat that?” • Quality Assurance: “Does this positive test meet the NHSN definition of a VAE- Do I need to report this case?”	• Laboratorian: “Should clinicians be using these confusing results?” • AS Team: “Do clinicians realize treating these results may not help, and may cause harm to the patient? Is reporting this result reinforcing this test is useful?” • Clinicians: “If the result is reported it is relevant, right? I did not know the specimen was a poor sample. How do I weigh pre-test probability?” • Quality Assurance: “Are respiratory cultures impacting our VAP or CLABSI metrics? Is VAP and VAE reporting standardized at our institution? What is the resource utilization for these tests?”	• Laboratorian: “Could we reject samples that are likely to grow normal flora like we do with expectorated sputum samples?” • AS Team: “Can we change who we test or how a specimen is processed and reported to make the results more useful?” • Clinicians: “Can I receive only information that is relevant and actionable?” • Quality Assurance: “Could we make an algorithm to clarify pre-test probability, testing, processing, and resulting?”	• Laboratorian: Change lab protocols and reporting to implement selective testing criteria for specimens, with mechanism to request culture for clinicians (override) • AS Team/Clinicians/Quality Assurance: • Provide education and clinical decision support regarding when a patient has high pre-test probability for bacterial respiratory infection and warrants testing. Change the EMR interface when ordering to include indications for a tracheal aspirate culture. Provide instruction how to obtain tracheal aspirate specimens.	• Laboratorian: Testing volume, time processing specimens, supplies/waste/resources saved, frequency of clinician requests to override AS Team: Rate of tests/ventilated-days, antibiotic days of therapy/ ventilated days • Clinicians/Quality Assurance: Number of intubated days per patient, rate of VAEs
Blood cultures	• Laboratorian: “This is a lot of work to identify and do susceptibilities on likely contaminants, sometimes these are send-outs.” • “Do clinicians understand that *mecA* means oxacillin-resistant, or that oxacillin susceptible also means cefazolin susceptible?” • AS Team: “When clinicians see “*Enterobacterales*” and *E. coli*” they may think there are two organisms.” • “Treatment of contaminants leads to a lot of vancomycin use” • Clinicians: “I am not familiar with the rapid results reported in this blood culture, I will wait for the culture to be finalized before stopping any antibiotics” • Quality Assurance: “We send a lot of blood cultures, there seems to be a lot of contaminants.”	• Laboratorian: “Why do we report it this way? Are we able to do this differently, or is this dictated by our testing platforms?” • AS Team: “These new PCR platforms lose value unless we connect old knowledge with new technology. Connecting the genetic targets to the organism and resistance implications is hard and takes practice, can we make this easier?” • Clinicians: “Could I safely decrease the number of blood cultures I order?” • “Is it possible to simplify the blood culture results to help me interpret what this result means? • Quality Assurance: “Does this meet the definition of a CLABSI, even though it is likely a contaminant?”	• Laboratorian: “We could simplify these results.” • AS Team: “Can we aid in interpretation and treatment choices in real time? Can we help the lab optimize how results are displayed? Could we stop sending these when not needed to prevent treating patients with skin contaminants with vancomycin?” • Clinicians: “Can the results use microbiology terms I am familiar with? • Quality Assurance: • “Could we create an algorithm for when to send a blood culture?” • “Would having dedicated staff to obtain blood cultures decrease contamination rates?	• Laboratorian: Implement improved result reporting in the EMR, and provide updates that cultures are negative at 24 and 36 hours to help clinicians interpret results at these important clinical time points. We could also add reminder that blood cultures are monitored around the clock and are negative unless reported positive. • AS Team: • Provide real-time result interpretation support and antimicrobial selection guidance when cultures return positive. • Clinicians/ Quality Assurance: Develop education and decision support on judicious blood culture ordering, creation of data-driven algorithms	• Laboratorian: • Volume of calls for clarification and identification of susceptibilities for contaminants. • AS Team: Vancomycin days of therapy, time to optimal therapy in bacteremic patients • Clinicians: Treatment of fewer contaminant blood cultures, evaluate for concerns for delay to treatment • Clinicians/ Quality Assurance: positive blood cultures per central line days, rate of contaminated blood cultures, rate of hospital onset drug-associated acute kidney injury, number of patients admitted after ED cultures that were potential contaminants
Gastrointestinal panels (GIPs)	• Laboratorian: “There are a lot of GIPs ordered, and we frequently see them on the same patients” • AS Team: “We are seeing lots of treatment and retesting” • Clinicians: “This *C. difficile* test result was released to the parents. It will be difficult to explain why we’re not treating” • Quality Assurance: “There seems to be a lot of positive GIPs for *C. difficile*”	• Laboratorian: “Is some of this testing unnecessary? Could we change our process for accepting and reporting samples?” • AS Team: “Sensitive tests can detect colonization- that seems to be reinforcing unnecessary testing and treatment” • Clinicians: “I order a GIP looking for other organisms. I can’t help it *C. difficile* is reported” • Quality Assurance: “Does our institution have a problem with transmissions? Or is over-testing part of the problem?”	• Laboratorian: “Can we build specimen quality measures into the EMR and suppress some results?” • AS Team: “Change will take a lot of education, this has become a vicious cycle. We can educate on rounds” • Clinicians: “Is it possible to selectively report what is significant? Could we decrease confusion by not reporting colonization?” • Quality Assurance: “Testing only when pre-test probability is high will give better information on our HAIs”	• Laboratorian: Laboratory selective testing criteria (Bristol stool criteria, no patient laxative use), selective reporting based on pathogen and age, mechanism to see suppressed results by request. • AS Team/Clinicians: • Support clinicians to interpret positive GIP, particularly for potentially colonizing organisms like *C.difficile*. • Clinicians: Provide clinician perspective for education needed and how best to implement restriction criteria. • Quality Assurance: Implement order restrictions in the EMR (eg, cannot proceed if patient is actively treated with laxatives, or has had the same test within 3 days)	• Laboratorian: Sample quality, number of specimens tested, number of override requests • AS Team: Use of oral vancomycin • Clinicians: Number of patients that progress to fulminant disease • Quality Assurance: Hospital-onset *C.difficile* rates
Urine testing	• Laboratorian: “We are often asked to do identification and susceptibilities on polymicrobial cultures, or to culture low quality specimens (eg, urine from cotton balls)” • AS Team: “Many patients do not get UAs, and polymicrobial cultures are not interpretable, however, often are treated” • Clinicians: “Obtaining a urine sample in a child is difficult and often distressing to the family. It makes sense to obtain one urine specimen for UA and culture” • Quality Assurance: “I’m seeing positive urine cultures in patients with indwelling urine catheters, is this a CAUTI or over-testing?”	• Laboratorian: “Could we make this test more useful and results more meaningful? Reporting bacteria from likely contaminated samples could be easily misinterpreted” • AS Team: “How many UA-negative, culture-positive urines do we need to treat to benefit or to harm one patient?” • Clinicians: “I understand I should only send urine cultures if the UA is abnormal, but manually separating these steps does not fit nursing and clinician work flow. Could we automate this process? ” • Quality Assurance: “Can we assess pre-test probability better, and if indicated, send a better sample?”	• Laboratorian: “Is it possible to selectively culture only specimens with a positive UA, from an appropriate sample?” • AS Team: “Could we update the clinical pathway/EMR to reflect utility of UA data? Can we help the lab with adoption by clinicians of any changes?” • Clinicians: “Could we have one process for sample collection and another for screening?” • Quality Assurance: “Could criteria in the EMR help drive appropriate testing?”	• Laboratorian: Conditional ordering: “Culture if urinalysis positive” or “Culture regardless of urinalysis” options, laboratory selective culture criteria based on specimen type (and urinalysis result) • AS Team/Clinicians: Education on urine specimen evidence in children, guideline development • Quality Assurance: Reflex urine cultures, education on specimen collection	• Laboratorian: Assess number of specimens tested and percent with actionable results • AS Team: Number of children treated for in-hospital acquired UTIs and associated antibiotic days of therapy • Clinicians: Assessment of missed cases (development of pyelonephritis), cases of adverse events after treatment of a UTI, Research implications of not treating UA-negative, culture-positive specimens • Quality Assurance: CAUTI rates, pathway compliance with testing and sampling

## Endotracheal aspirates

*Background state prior to diagnostic stewardship*: Endotracheal aspirate cultures (EACs) are commonly used in the evaluation of pediatric patients with artificial airways suspected to have pneumonia or tracheitis.^9^ However, EACs may demonstrate bacteria that are present in the airway but not causing infection, limiting their overall specificity in diagnosis.^10^ Adult centers may use bronchoalveolar lavage (BAL) or mini-BAL more often than pediatric settings however, across the age spectrum, national guidelines promote EACs over invasively obtained samples.^11^ Ordering, processing, and reporting of EACs are highly variable,^9^ and contribute to antimicrobial prescribing.^12,13^ Processing these samples requires laboratory time and resources, as these cultures often grow multiple organisms that would require significant resources to perform speciation and susceptibility testing. Typically, most bacterial growth in cultures is reported, even when the result likely represents colonization. Compounding this issue, many electronic medical records (EMRs) report any positive test result with emphasized text (eg, red, capitalized, highlighted, use of exclamation marks) and may add wording indicating an “abnormal result.” These results and signals reinforce perceived diagnostic value of the test result, nudging clinicians toward treatment. Respiratory infections have overlapping features with other pathology, and even a seasoned clinician who understands the low specificity of an EAC may have difficulty ignoring an “abnormal” result, particularly when the patient is very ill, there exists diagnostic uncertainty, and the result is released to the family.^14,15^

*Optimal state after diagnostic stewardship*: There are multiple moments of differing understanding and priorities by healthcare workers along the process of a diagnostic test that allow opportunity for collaboration to assure the test adds value (Table 1). Two clinician-based targets for quality initiatives targeting EACs with success include education to consider pre-test probability and standardization of specimen collection.^16–18^ A laboratory-based approach is pre-screening specimens for quality to reduce the processing of specimens that are likely to be negative or uninterpretable. Laboratories can enact conditional culturing based on Gram stain features, and/or require order clinical indications for testing.^19–21^ Children’s Hospital Colorado’s laboratory enacted selective culture criteria, and now does not culture specimens that are likely to be low diagnostic value (ie, specimens with a Gram stain that is negative for organisms, positive for three or more bacterial morphologies, and/or positive for epithelial cells). These scenarios exemplify the role of the laboratory to decrease test results that may be non-specific and risk misinterpretation by clinicians. AS can support these types of changes, by listening attentively for stakeholder concerns, liaising between the laboratory and clinicians, educating about the enacted changes, and assessing results, including consideration of impact on antibiotic prescribing and balancing measures (eg, ventilator-associated events, ventilator-days).

## Blood cultures

*Background state prior to diagnostic stewardship*: Blood cultures are often overutilized for common pediatric hospital conditions requiring admission.^22^ The time for a blood culture to grow, for an organism to be identified, and initial susceptibility data to be known is significantly less than in the past.^23,24^ Clinician understanding of the laboratory methods in this fast-moving field are sometimes outdated; this results in clinicians 1) holding on to the 48–72 h “rule out” (which is reinforced by automated results appearing in the EMR only at 24-h intervals), 2) distrusting results of multiplex polymerase chain reactions (PCRs) or not considering them “final” enough to target antimicrobial therapy, and 3) misinterpreting reported results, particularly when the laboratory reports a list of gene targets without an interpretation (eg, “detected: enterobacterales, *Klebsiella pneumoniae*, *ctx-m*” rather than the interpreted “ESBL-producing *K. pneumoniae*”). The antimicrobial steward can bring laboratorian observations to clinicians, and bring clinicians’ reflections to the laboratory, work with quality assurance teams to explore optimal processes, reinforce appropriate indications for blood culture collection, and optimize EMR interfaces to embrace the full potential of new platforms for rapid blood culture identification.

*Optimal state after diagnostic stewardship*: In terms of DS in clinician decision-making, the literature demonstrates the low utility of blood cultures in various clinical syndromes, including uncomplicated community acquired pneumonia, skin and soft tissue infections, surveillance for thermoregulated patients, and repeat cultures in patients with gram-negative bacteremia of urinary source.^22,25,26^ In addition, clinical algorithms can be used to decrease blood culture testing when the pre-test probability is low, particularly during blood culture bottle shortages^27^, and thus avoid significant cost, resource utilization, patient discomfort, and the adverse implications of a contaminant for a patient.^28,29^ AS ideally works with clinicians and quality assurance teams to implement better ordering practices up front, but even after a blood culture grows, the steward is an essential resource in supporting the interpretation and reaction to a test through real-time decision support and up-to-date guidance. Laboratories play an essential role in optimizing reporting of results into language that is easily interpreted by clinicians. Notably, increased impacts of novel rapid molecular diagnostics such as multiplex PCR and MALDI-TOF are realized by partnering with AS,^30–32^ as reinforcement and education within specific patient-contexts may be key to avoiding unnecessary antibiotic prescribing. Opportunities for the care triangle to explore include AS relaying positive results in real-time, including interpretation of results and resistance markers, antimicrobial recommendations, explanation of unfamiliar species names, and prevention of readmissions^32^ and treatment of likely contaminants. Antimicrobial stewards may be the first to realize trends in culture results/contaminants that should be reported to IPC for investigation. To garner local support, local evaluation is often needed, either to enact change, or as follow-up to change for reassurance regarding patient safety.

## Gastrointestinal molecular panels

*Background state prior to diagnostic stewardship*: Prior to multiplex PCR testing of stool, gastroenteritis was a clinical diagnosis or based on less sensitive, more complicated testing (eg, stool culture, ova and parasite testing, viral electron microscopy, ELISA). With the implementation of multiplex PCRs (gastrointestinal panel, GIP), identification of organisms increased, but in many circumstances, this added uncertainty around the clinical significance of positive testing.^33,34^ *Clostridioides difficile* testing highlights this conundrum, as pediatric patients are known to be colonized early in life.^35^ Although some clinicians understand this epidemiology, others may still interpret a positive *C. difficile* result as indication for treatment. Infection preventionists are required to track and report HAIs, which can be falsely inflated due to non-selective testing criteria and result reporting. Other GIP results, such as the various targets for *Escherichia coli* spp., are also observed to cause confusion.

*Optimal state after diagnostic stewardship*: Creative DS solutions are needed to curb unnecessary multiplex PCR testing and resultant antimicrobial overuse. Potential DS solutions include patient risk stratification,^36^ EMR-based conditional ordering, and laboratory specimen selective testing based on stool quality, recency of testing, and laxative use. One quality initiative coupled education with EMR conditional ordering to reduce rates of low-value stool testing.^37^ Selective reporting of results from the laboratorian side may be preferable to trying to teach clinicians to “ignore” certain results. For example, *C. difficile* can be selectively reported only when specifically ordered, and/or only in children over one year of age. In some adult centers, *C. difficile* is only reported if a reflex toxin assay is positive.^38,39^ These potential solutions all require significant collaboration by those in the care triangle (Figure 1), particularly for de-implementation efforts if testing has become a very common institutional practice contributing to over-diagnosis and treatment.^40^

## Urine testing

*Background state prior to diagnostic stewardship*: In pediatrics, there are significant DS opportunities around the diagnosis and treatment of urinary tract infections. Progress is hampered by lack of gold standard definitions, lack of high-quality data, and propagation of certain dogma. To avoid the need to obtain another sample, many pediatric clinicians send a culture in tandem with a urinalysis (UA), rather than sending the culture only after an abnormal UA.^41^ For laboratory diagnostics, the definition of a positive UA, or even a positive culture, is not historically well developed,^42,43^ leading clinicians to question the utility of a UA in neonates in particular. Recent work challenges this belief,^44–46^ including supporting the strong positive and negative predictive value of a UA for bacteremic pyelonephritis.^44^ Another misconception is that urine is a sterile fluid,^47^ and identifying bacteriuria is abnormal. Converse to adult patients,^48^ efforts to prevent treatment of asymptomatic bacteriuria (ASB) in pediatric patients are lacking, despite studies demonstrating ASB may be present in 1%–10% of healthy children^49–51^ and 95% of cases may resolve without treatment,^52^ consistent with adult data.^53–55^ Although we have some understanding how many children with a negative UA may have a positive culture,^56^ our understanding of the number of such patients needed to treat to benefit one (eg, prevent progression to serious infection) vs number needed to treat to harm one is paramount to DS of urine cultures in pediatrics. Laboratorians are often asked to culture, identify, and perform susceptibility testing on specimens that had a negative or no UA, were obtained via nonsterile collection (eg, cotton ball, indwelling catheter), and/or are growing multiple colony types suggestive of contamination. Hospitals may be required to report these results as HAIs. Stewards observe unnecessary treatment of these “infections” and downstream adverse effects.

*Optimal state after diagnostic stewardship*: The UA should be used in all age groups to guide decisions on whether to culture a urine specimen based on high negative predictive value.^44,45^ In children with a negative UA, it is likely appropriate to recommend watchful waiting in the majority of cases. Clinicians and laboratorians can collaborate on DS efforts to reduce inappropriate urine culturing. Using the UA to determine which specimens should be cultured could be done at the level of clinician or, alternatively, at the level of the laboratory; reflecting from a workflow perspective, the second option has many benefits in pediatrics, given the need to perform catheterization to obtain a sample.^48,57^ A clinician-driven option is to only catheterize a child if the UA is positive.^58–60^ A collaborative solution could include conditional ordering in the EMR, eg, “culture only if UA abnormal,” or “culture even if UA abnormal” (to accommodate special situations like neutropenic hosts). A lab-based initiative could require the clinician to order a culture, with the laboratory performing the UA and doing the culture only if it is positive. All would decrease culturing of UA-negative specimens. Such changes are likely to benefit from AS and IPC to aid in enactment, understanding of result implications, and monitoring of impact and balancing measures.

## Harnessing other potential opportunities and following through

Although these examples illustrate the collaborations between clinicians, laboratorians, and quality assurance teams, additional opportunities for antimicrobial stewards in DS require a system of prioritization. In all cases, local stakeholders are vital, and the antimicrobial steward promotes observation, reflection, exploration, enactment and evaluation while pivoting between members of the care triangle. For example, if there is a clear opportunity for an improvement, but there are no obvious invested stakeholders, the steward may need to focus on laying groundwork for change and seeding the idea across the hospital until another interested party emerges and the project can move to the Exploration and Enactment stages. Conversely, if stakeholders approach the antimicrobial steward seeking change, supporting the interest quickly is key to harnessing momentum.

Stewardship of certain tests may be particularly high value such as 1) uncommon but expensive tests (eg, untargeted DNA sequencing^61,62^), 2) tests developed for adult patients with unclear application for pediatric patients (eg, bone and joint infection multiplex PCRs designed for infection of prosthetic joints in adults), and 3) tests ordered very commonly that lack diagnostic specificity for the infectious process or do not benefit management decisions. In infectious diseases, there are many examples that fall into this third category: eg, inflammatory markers, chest radiographs, viral blood PCRs, IgM tests with low specificity, and fungal markers. Beyond focus on a specific test, consideration of particular clinical scenarios where testing may be over utilized can improve value, for example, pan-cultures in febrile intensive care unit (ICU) patients, surveillance cultures in thermoregulated patients, optimal testing for patients with respiratory symptoms (eg, shift from broad multiplex PCR panels to narrower PCR panels/no PCRs and point of care testing).^63–67^

The final moment of AS-DS synergy is following through on initiatives to evaluate efficacy and potential harm (Table 1).^68^ Desired outcomes will vary by initiative. Balancing measures (potential harm) can include length of stay, return or escalation to intensive care, readmission, changes in HAI incidence, and progression to more serious infection. Non-clinical outcome measures may be considered as well, such as an increase in use of an alternative tests, clinician time, and patient satisfaction (eg, patients may expect testing and it takes more time to explain why they may not benefit from a test). Measurement of both desired and undesired outcomes is important to solidify change, iterate unsuccessful initiatives, and reassure clinicians to further improve patient care and disseminate optimal practices.

## Cost

DS programs will require personnel and system resources that incur costs. Although this may require financial support, the benefits of avoiding problematic diagnostic testing will justify the cost. With the overabundance of information a clinician must sort through every day, AS and DS need to synergize to enhance the utility of diagnostic tests. The overriding goal of this DS-AS synergy is to increase the value of care, through optimizing test ordering, implementation, and reporting. Although some tests may add cost, if they improve outcomes this may add value; conversely, some tests do not improve outcomes, result in actionable care, may add harm, and are costly and/or frequent, lowering value. There are costs to the hospital, the patient, and society. Hospital costs may be measurable, or more indirect. Examples include costs of supplies and equipment, costs for person power to collect, process and report testing, costs of treatment^69^ and costs of extended hospital stays. The downstream impacts of unfavorable HAI metric reporting can include losses to reimbursement, or fewer admissions due to impacts on reputation. The additive values are substantial; as an example, a single pediatric *C. difficile* case may cost $93,000.^70^ Potentially avoidable patient and family costs include the direct cost of the testing and treatment and longer time away from work or school, and risk for experiencing an adverse drug reaction and associated care needs. Another potential harm to families that is difficult to measure is the psychologic strain or confusion when patients see positive results but may not understand how to interpret them. Costs to society include healthcare expenses, antimicrobial resistance, and environmental pollution.^71^

As an example of the latter, microbiology laboratories dispose of a large amount of waste daily from routine cultures; the costs, in terms of monetary, energy, and greenhouse gases, for disposal of this waste are considerable.^72^ Sources include autoclaving or incineration prior to disposal, transportation to processing areas, and methane emissions occurring from landfills. Adding to this is the antibiotic waste disposal associated with discarded unused anti-infectives, and the water contamination from administered, then excreted antibiotics. Understanding the true costs of diagnostic and antimicrobial overuse to our hospitals, our patients, and society provides the context needed for the best care and support for our programs.^73–75^ The downstream implications may be notable. As an example, if unnecessary blood cultures are prevented, this leads to less contaminant blood cultures in patients without true bacteremia, fewer repeated confirmatory blood cultures, fewer reported central line-associated bloodstream infections (CLABSIs), less treatment for bacteremia that was not real, fewer adverse impacts from unnecessary antimicrobials, like *C. difficile,* fewer resources to hospitalize and care for the patient, fewer supplies and personal protective equipment gear utilized, and less waste and environmental contamination. The recent commercial blood culture bottle shortage in 2024 may serve as a natural experiment to study the impact of reduced blood culturing from a patient outcomes, IPC, and environmental lens.

Identifying low value processes to target for improvement takes multidisciplinary observation and identification of common goals. The antimicrobial steward is uniquely placed to add to these observations, connect and reflect with interested parties, facilitate stakeholder exploration, and synergize the processes of enactment and evaluation. Although true for all ASPs, a large portion of the success of Handshake Stewardship stems from collaborative diagnostic stewardship efforts^76–78^ originating from in-person discussions during microbiology and stewardship rounds. As AS evolves, there is opportunity to expand AS programs to include DS in collaboration with clinicians, laboratorians, IPC, and quality assurance teams.
